# Direct Photocoagulation Guided by Merged Retinal Images for the Treatment of Focal Diabetic Macular Edema

**DOI:** 10.1155/2018/2401094

**Published:** 2018-03-12

**Authors:** Yoshihiro Takamura, Takehiro Matsumura, Shogo Arimura, Makoto Gozawa, Masakazu Morioka, YutakaYamada, Masaru Inatani

**Affiliations:** Department of Ophthalmology, Faculty of Medical Sciences, University of Fukui, Eiheiji-cho, Yoshida-gun, Fukui-ken 910-1193, Japan

## Abstract

**Purpose:**

To introduce a novel laser photocoagulation (PC) protocol named merged image-guided PC (MIG-PC), which included merging the images of the fundus, optical coherence tomography (OCT) map, and fluorescein angiography (FA). We compared the anatomical and functional results between MIG-PC and FA-guided PC (FG-PC) for the treatment of focal diabetic macular edema (DME).

**Method:**

We examined the treatment outcomes in 27 consecutive eyes treated with MIG-PC compared with 28 matched eyes treated with FG-PC. We identified the microaneurysms (MAs) located in the focal edema areas and ablated them using focal PC. Best-corrected visual acuity (BCVA) and retinal thickness (RT) measured using OCT were compared between the groups at baseline and 2, 4, 8, 12, and 24 weeks after treatment.

**Results:**

The foveal and perifoveal RT were reduced after treatment in both the groups, and the perifoveal RT in the MIG-PC group was significantly lower than that in the FG-PC group at 4 weeks and thereafter. BCVA in the MIG-PC group was significantly higher than that in the FG-PC group at 12 and 24 weeks. The numbers of laser spots (*p* = 0.0001), additional laser treatments (*p* = 0.0121), and intravitreal injection of ranibizumab (*p* = 0.0012) in the MIG-PC group were significantly lower than those in the FG-PC group (Mann–Whitney test).

**Conclusion:**

MIG-PC contributed to the improvement in BCVA and reduction in RT, number of laser shots required, and retreatment rates. Based on our data, MIG-PC can be recommended for the treatment of focal DME. This trial is registered with ID UMIN000030390.

## 1. Introduction

Diabetic macular edema (DME) is the most common cause of visual loss in patients with diabetes [1]. DME is generally differentiated into diffuse and focal DME, and microaneurysms (MAs) are associated with the pathogenesis of both these types. Typically, MAs are found in the area of thickened retina with a circinate ring of exudation [2]. The leakage of blood constituents from MAs into the retinal tissue results in the swelling of the retina. Focal laser photocoagulation (PC) for MAs is considered as the standard treatment [3, 4]. The potency of focal PC may be partially attributed to the closure of MAs. The Early Treatment Diabetic Retinopathy Study (ETDRS) showed that focal/grid PC reduced the risks of losing ≥3 lines of vision by approximately 50% for 3 years after treatment [5]. Based on the evaluation of prospective clinical trials, the mainstays in the treatment for DME have shifted to new modalities, such as the use of antivascular endothelial growth factor (VEGF) [6, 7]. However, several DME cases show a low response to anti-VEGF treatment, and repeated injections are required to maintain its therapeutic effects [8]. Direct PC aiming at MAs remains an important option in the treatment of extrafoveal DME.

In focal PC, the goal is to close MAs and stop the leakage, while avoiding retinal scarring. Focal PC is delivered with very low energy compared with panretinal photocoagulation (PRP); nevertheless, some enlargement of laser scarring may lead to the atrophy of the retinal pigment epithelium (RPE) and photoreceptor cells. If the laser scar is placed very close to the foveal center, visual acuity may be severely impaired. Therefore, ophthalmologists should pay attention to performing focal PC accurately with minimum number of shots and avoid overzealous treatment.

In focal PC for treating MAs, accurate information regarding the location of MA is important. On fundus examination, MAs and small hemorrhages can be recognized as tiny round red dots. In fluorescein angiography (FA), MAs and hemorrhages appear as hyper- and hypofluorescent spots, respectively, and are thus distinguishable. MAs located in the thickened area may be associated with DME pathogenesis. Recently, mapping images using spectral domain-optical coherence tomography (SD-OCT) has provided information regarding the degree and distribution of macular thickness [9].

In this study, we overlapped the images of the ocular fundus, FA, and OCT map of the eyes with DME. Using this novel protocol, we could visualize the location of MAs on macular lesions and apply direct focal PC to these MAs; we compared the anatomical and visual outcomes with those for the eyes treated with FA-guided PC.

## 2. Method

**Figure 1:**
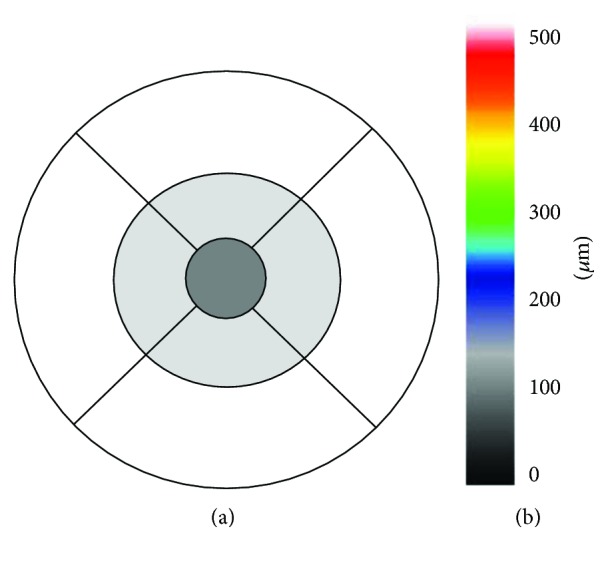
(a) Location of the three fields on the macular thickness map. Center: dark-gray field; inner ring: light-gray field; outer ring: white field. (b) A sample of the false-color topographic image of optical coherence tomography (OCT).

This retrospective comparative study adhered to the tenets of the Declaration of Helsinki and was approved by the University of Fukui Institutional Review Board. This study was registered with the University Hospital Medical Information Network Clinical Trials Registry (UMIN-CTR) of Japan (UMIN000030390). This study included 27 consecutive eyes with focal DME treated with FA-guided PC (FG-PC group) between November 2014 and December 2015 and 28 eyes with focal DME treated with merged images of fundus photographs and OCT maps (MIG-PC group) between January 2016 and May 2017 at the Fukui University Hospital. We enrolled patients (age, >20 years) diagnosed with type 2 diabetes mellitus with focal DME. In this study, focal DME was defined as the leakage from MAs on FA and focal swelling in retinal topography on OCT. Exclusion criteria included a history of prior vitrectomy in the study eye, history of anti-VEGF injections received within 3 months, history of PRP within 6 months, and the presence of medial opacity, such as severe cataract, corneal opacity, or vitreous hemorrhage. All patients underwent comprehensive ophthalmic examinations, including visual acuity testing, slit-lamp biomicroscopy, measurement of intraocular pressure (IOP) using the Goldmann applanation tonometer, and dilated fundoscopic examination. Color fundus photographs and FAs were captured using a Kowa VX-10i fundus camera (Kowa Ltd., Nagoya, Japan) and Spectralis Heidelberg Retinal Angiograph+OCT (Heidelberg Engineering, Heidelberg, Germany), respectively, at baseline before laser PC. Using Cirrus OCT (Carl Zeiss Meditec, Dublin, CA), we measured the retinal thickness (RT) using the fast macular thickness protocol at baseline and 2, 4, 8, 12, and 24 weeks. The analysis of RT reconstructed a false-color topographic image displayed with numerical averages of the thickness values for all nine map sectors defined by ETDRS (Figure 1(a)). The scanned areas were segmented into the center, inner, and outer rings, with diameters of 1, 3, and 6 mm, respectively. We calculated the average RT using four corresponding sections in the inner and outer rings and ratio of the edematous areas in the macular area. The number of pixels in highly swollen areas, indicated by the white area on OCT color maps, representing >500 *μ*m, was calculated using Adobe software (Photoshop CS6 Extended, Adobe Systems Inc., San Jose, CA) and divided by the pixels in the macular area indicated by black lines in Figure 1(b).

### 2.1. Treatment Protocols

**Figure 2:**
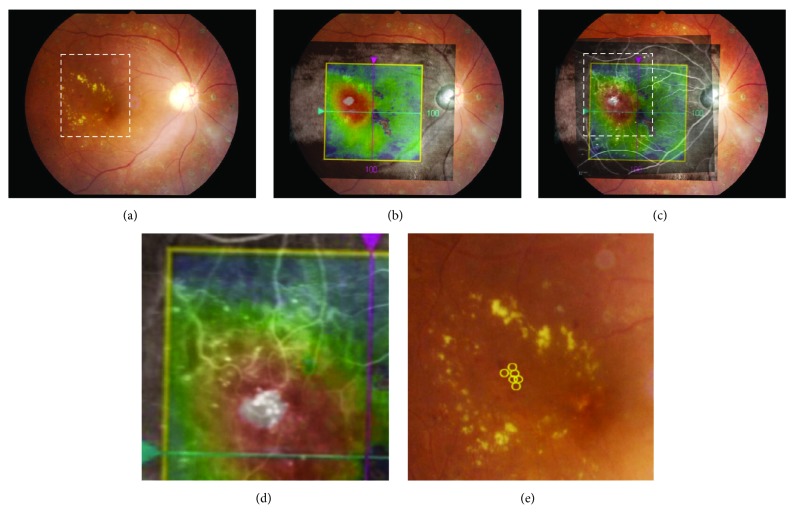
Steps for marking the location of microaneurysms (MAs) in the merged image-guided photocoagulation (MIG-PC) method. (a) Ocular fundus photograph shows focal diabetic macular edema accompanied by a hard exudate ring. (b) OCT map is overlaid on the fundus photograph. (c) Image of fluorescein angiography (FA) is merged with the fundus photograph and OCT map. (d) High-magnification image corresponding to the rectangular area is indicated with white-dashed line in Figures 2(a) and 2(c). (e) MAs are marked on the ocular fundus as yellow circles.

To determine the location of MAs in focal DME, images of the OCT map and FA were superimposed and aligned to fundus photographs according to retinal landmarks, such as retinal vessels and the optic disc, using Adobe Photoshop (CS2, Adobe Systems Inc.). To complete this process, images were enlarged and placed as needed. The layers of OCT and FA images became transparent when opacity was set to 70% and 50%, respectively, so that the underlying images of MAs in the ocular photographs showed through (Figure 2). We marked the MAs located in the focal edema expressed by red or white colors with a circle on the ocular photograph (Figure 2(e)). Finally, fundus photographs marked with MAs were displayed on the computer screen besides the patient and used as a guide while performing laser treatment. To perform direct PC, we referred to FA images alone to identify the location of MAs.

Laser PC was performed following pupillary dilation and instillation of topical anesthesia using H-R Centralis® (Volk Optical Inc., Mentor, OH, USA) by a single retina specialist (YT). We used a PASCAL photocoagulator (Optimedica Corporation, Santa Barbara, CA, USA) with a frequency-doubled neodymium-doped yttrium aluminum garnet solid-state laser with a wavelength of 577 nm. The laser parameters were as follows: (1) spot size of 60 *μ*m, (2) pulse duration of 20 ms, (3) single spot, and (4) burn intensity of 100–150 mW, which was increased until a gray/white lesion was attained. Patients were eligible for additional PC if edematous areas indicated by white color in a false-color topographic image were still present. Additionally, patients were eligible for the intravitreal injection of ranibizumab (IVR) (Lucentis, Genentech Inc., San Francisco, CA, USA), anti-VEGF drugs if the central RT (CRT) was >350 *μ*m at 8, 12, and 24 weeks after the initial treatment.

### 2.2. Statistical Analysis

We performed statistical analyses using JMP (SAS institute Inc., Tokyo, Japan). We used Bartlett's test to examine equal variances across samples, followed by assessing the statistical significances between the groups using the Mann–Whitney test. Furthermore, statistical analyses were performed using the Wilcoxon signed-rank test (pre- and posttreatment data in the same group). Values were expressed as the mean ± standard deviation. Differences were considered statistically significant at *p* < 0.05.

## 3. Results

**Figure 3:**
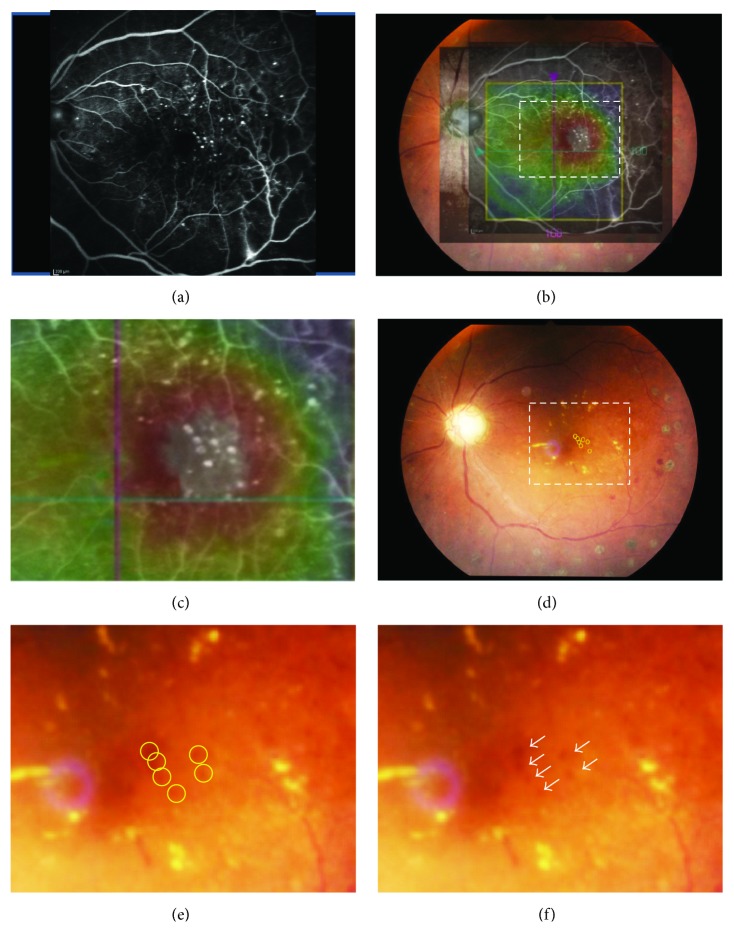
A sample of the MIG-PC method. (a) FA image shows the MAs as dots of hyperfluorescein. (b) The OCT map image is merged with the FA image and fundus photograph. (c) High-magnification image corresponding to the rectangle area is indicated with white-dashed line in Figures 3(b) and 3(d). MAs were noticed in the thickened retinal area. (d) MAs are marked as yellow circles on the ocular fundus photograph. (e) High-magnification image corresponding to the rectangle area is indicated with white-dashed line in Figure 3d.

**Figure 4:**
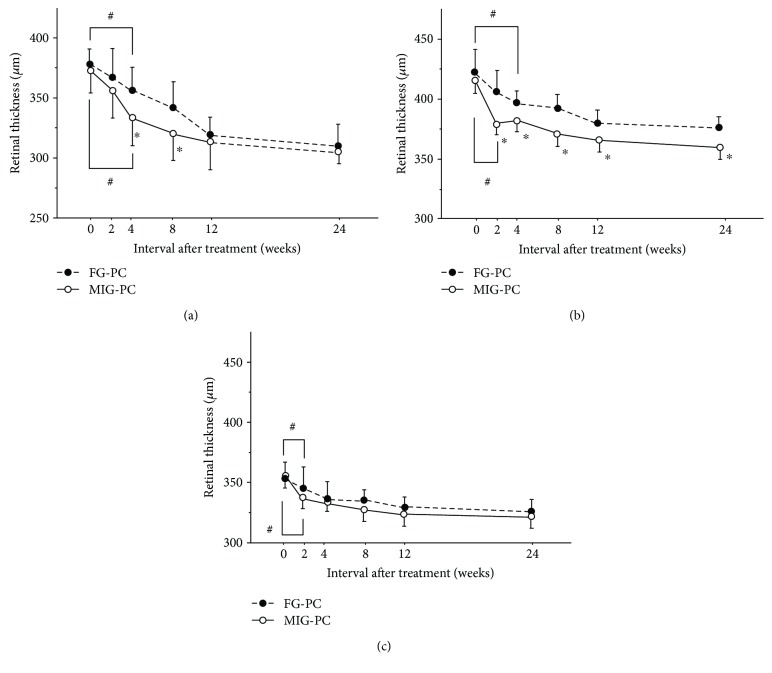
Changes in retinal thickness (RT) in the center (a), inner ring (b), and outer ring (c) after fluorescein angiography-guided focal photocoagulation (FG-PC) or merged image-guided PC (MIG-PC). Data represent the mean ± standard error (SE). ^#^*p* < 0.05 (compared with RT at PC initiation) and ^∗^*p* < 0.05 (MIG-PC group versus FG-PC group).

**Figure 5:**
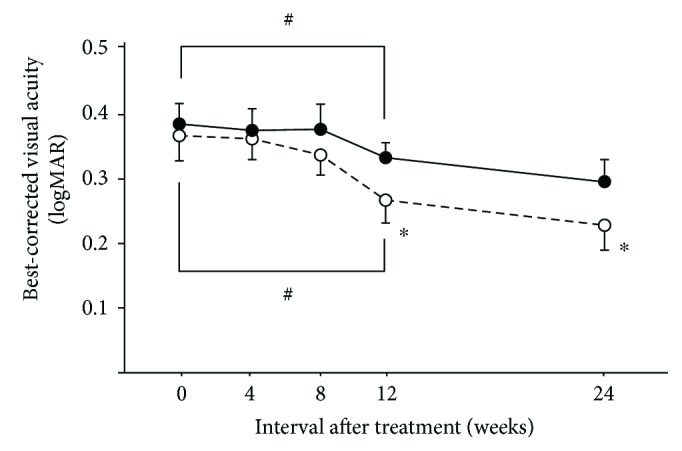
Changes in best-corrected visual acuity (BCVA) (logarithm of the minimum angle of resolution (logMAR)) after focal laser treatment in the MIG-PC and FG-PC groups. Data represent the mean ± standard error (SE). ^#^*p* < 0.05 (compared with RT at PC initiation) and ^∗^*p* < 0.05 (MIG-PC group versus FG-PC group).

We analyzed 28 eyes in the MIG-PC group and 27 in the FG-PC group. There was no significant difference in the baseline characteristics, visual acuity, and CRT between the groups (Table 1).  Figure 3 shows a sample case of the MIG-PC group. The merged images indicated that some MAs visualized by FA (Figure 3(a)) were located in the highly thickened areas (Figures 3(b) and 3(c)). These MAs are marked by yellow circles (Figures 3(d) and 3(e)). The ratio of the swollen area to the macular area was 28.1 ± 8.3% and 25.2 ± 6.9% in the MIG-PC and FG-PC groups, respectively (Table 2); the difference between the groups was insignificant. In the central area, a significant decrease in CRT was observed in both the groups; at 4 weeks and thereafter, CRT in the MIG-PC group was significantly lower than that in the FG-PC group at 4 and 8 weeks after laser treatment (Figure 4(a)). In the inner ring, a significant decrease in RT was found at 2 weeks in the MIG-PC group, but only at 4 weeks in the FG-PC group (Figure 4(b)). RT in the MIG-PC group was significantly lower than that in the FG-PC group at 2, 4, 8, 12, and 24 weeks after surgery. In the outer ring, a significant decrease in RT was observed in both the groups at 2 weeks after the treatment; no significant difference was found between the groups (Figure 4(c)). Visual acuity was significantly improved in both the groups at 12 and 24 weeks from baseline. BCVA in the MIG-PC group was significantly higher than that in the FG-PC group at 12 (*p* = 0.026) and 24 (*p* = 0.032) weeks (Figure 5).

The data of other parameters in the treatment are shown in Table 2. The number of additional laser treatments was 0.87 ± 0.62 in the FG-PC group and 0.42 ± 0.58 in the MIG-PC group; the difference was significant (*p* = 0.0121). Also, the number of IVR was greater in the FG-PC (0.91 ± 0.59) than in the MIG-PC group (0.35 ± 0.56; *p* = 0.0012). The difference between the average number of laser spots in the initial laser treatment (26.5 ± 9.2 in the FG-PC group and 16.4 ± 6.7 in the MIG-PC group) was significant (*p* = 0.0001).

## 4. Discussion

Targeting MAs using focal PC is a strong tool for improving macular swelling [3]. However, accurate focal laser treatment is technically difficult, and the failure of the procedure results in the presence of DME. In this study, we introduced a novel focal PC protocol guided with the merged images of FA, OCT map, and ocular fundus photography, named MIG-PC. Based on our data, focal MIG-PC resulted in the rapid reduction of perifoveal RT, indicating the superiority of MIG-PC compared with FG-PC. Based on FA findings, MAs could be defined as dots with hyperfluorescence, and their location could be determined in reference to the path of the main vessels. However, the positioning of existing MAs between large numbers of branching vessels is challenging. Using MIG-PC, we can utilize not only the path of blood vessels but also the hard exudate and hemorrhages and dot hemorrhages in determining the location of the MAs. Accurate ablation of MA with the aid of the merged images may thus contribute to better treatment outcomes.

The leakage from MAs which are located within the highly thickened retinal areas is probably responsible for the formation of focal edema [10]. In MIG-PC, we targeted the MAs located in the thickened areas. On the other hand, as FA does not provide us information regarding the geographical relationship between MAs and focal macular areas, MAs not only within but also surrounding the focal edema may have been photocoagulated in the FG-PC group. Although the width of the edematous areas was similar, the number of PC shots in the MIG-PC group was significantly lower than that in the FG-PC group. A suitable direct PC can achieve the closure of MAs, leaving cells in retinal layers intact. However, excessive thermal burns sometimes result in the destruction of the photoreceptor cells and RPE, leading to permanent vision loss [11]. Based on our data, MIG-PC is beneficial in decreasing the number of laser spots and thus preventing the onset of laser-related complications.

In the MIG-PC group, we photocoagulated MAs located in the swollen areas. MAs surrounding the edematous areas were not ablated even if they showed focal leakage in FA. Nevertheless, focal DME was improved more rapidly in the MIG-PC group than in the FG-PC group. Thus, MAs surrounding the focal edema may not be responsible for retinal swelling. Previous reports have shown that some MAs with dye leakage by FA did not completely overlap with retinal thickening on OCT [12–15]. The combination of FA and OCT map is informative in determining the MAs that are responsible for retinal swelling and treating the focal DME.

In this study, the numbers of IVR (anti-VEGF drug) and direct PC shots in the MIG-PC group were significantly lower than those in the FG-PC group. These data indicated that MIG-PC may be superior than FG-PC in terms of both the retreatment rate and overall injection burden. Minimizing the frequency of IVR would contribute to reducing the risks of cerebral infarction, endophthalmitis, and retinal detachment. In the retreatment criteria of our study, additional IVR was allowed at 8 weeks and thereafter. The difference in CRT between the groups was insignificant at the same time points, possibly due to the rapid therapeutic effects of retreatment. MIG-PC not only enhanced the decrease in CRT but also improved the final outcome of BCVA. As a significant improvement in BCVA followed the reduction in CRT, it is likely that the improved visual outcome resulted from the earlier recovery from DME.

In conclusion, our study reported MIG-PC as a new approach to performing focal laser treatment for focal DME. MIG-PC has some benefits over the conventional methods, specifically the increased accuracy in photocoagulating MAs and a reduced need for retreatment. MIG-PC may accelerate and enhance the improvement of visual acuity and DME achieved using conventional focal laser treatment based on FA images alone.

## Figures and Tables

**Table 1:** Baseline characteristics at the time of registration.

	MIG-PC group^a^ (*n* = 28)	FG-PC group^b^ (*n* = 27)	*p* value
Mean age (years)	65.3 ± 6.3	63.6 ± 7.2	0.47^a^
Mean hemoglobin A1c (%)	7.2 ± 0.3	7.5 ± 0.4	0.42^a^
Insulin therapy	15 (53.6%)	13 (48.1%)	0.58^a^
Left eye : right eye	12 : 14	14 : 13	0.64^b^
Gender (male/female)	16/12	15/12	0.57^b^
Mean duration of DM (years)	13.3 ± 2.3	12.6 ± 2.1	0.48^b^
Mean serum creatinine	2.11 ± 0.32	2.04 ± 0.22	0.38^a^

^a^Mann–Whitney test; ^b^chi-square test; DM: diabetes mellitus; MIG-PC: merged image-guided photocoagulation; FG-PC: fluorescein angiography-guided photocoagulation.

**Table 2:** Data relating treatment.

	MIG-PC group^a^ (*n* = 28)	FG-PC group^b^ (*n* = 27)	*p* value
Edematous areas (%)	28.1 ± 8.3	25.2 ± 6.9	0.24^a^
Injection of ranibizumab (*n*)	12.6 ± 2.1	26.5 ± 9.2	0.012^a^
Laser shots (*n*)	0.72 ± 0.58	1.87 ± 0.62	0.00012^a^
Additional laser (*n*)	1.25 ± 0.56	2.41 ± 0.49	0.00001^a^

^a^Mann–Whitney test; ^b^chi-square test; MIG-PC: merged image-guided photocoagulation; FG-PC: fluorescein angiography-guided photocoagulation.
